# Cultural dynamics influencing decision-making during the COVID-19 pandemic: the Italian case

**DOI:** 10.3389/fpsyg.2024.1294190

**Published:** 2024-04-25

**Authors:** Sara Costa, Giuseppe Carrus

**Affiliations:** Department of Education, Roma Tre University, Rome, Italy

**Keywords:** COVID-19, cultural dynamics, decision-making, qualitative thematic analysis, collectivism-individualism

## Abstract

The COVID-19 pandemic has brought about significant changes to the life of most individuals, worldwide. This study explores the cultural factors influencing decision-making during the pandemic, and is part of the CORNER Project, funded by the Research Council of Norway, aimed at understanding institutional response in the early phases of the Covid-19 emergency in Norway, Sweden, and Italy. Semi-structured interviews with key political-administrative leaders in Italy (*N* = 35) were conducted and content-analyzed, allowing the identification of the underlying cultural dynamics that played a role in these decisions. Thematic analysis was used to assess the influence of cultural factors on the crisis management and early reaction of institutions and citizens to the spread of Covid. In line with previous studies, and as expected, the individualism vs. collectivism dimension can explain differential health outcomes during the outbreak. In this paper we argue that individualism–collectivism cultural values can also play a pivotal role in public compliance with Covid-19 restrictions, and psychological responses during the pandemic.

## 1 Introduction

The COVID-19 pandemic has brought about significant changes to the way of life for many individuals. Governments around the world have responded to the crisis in various ways, implementing different strategies to maintain economic and social stability. The decision-making processes in addressing the COVID-19 pandemic were likely influenced by a variety of factors, with culture being a crucial determinant. According to scholars, the cultural perspective, particularly the classical collectivism vs. individualism dimension, significantly impacted the implementation of various measures, such as social distancing, mask use, self-quarantine, and city lockdowns (Xiao, 2021).

Culture can be defined as a shared system of meaning that shapes individuals’ fundamental psychological processes and influences their understanding of the world (Triandis, 2001). Two key dimensions of cultural variation are individualism and collectivism, which frame how individuals perceive reality and prioritize either the individual or the collective (Hofstede, 2001). These cultural values significantly impact people’s behavior, especially in response to novel challenges like the COVID-19 pandemic (Travaglino and Moon, 2021). In this context, both individualistic and collectivistic tendencies are expected to exert significant influence on people’s behaviors and choices.

Collectivism and individualism are usually defined as cultural perspectives that reflect different attitudes towards the relationship between individuals and groups, and the degree to which certain cultures emphasize independence versus interdependence (Hofstede, 2011; Park et al., 2016). The Hofstede’s model presents perhaps the most widely known and cited discussion of the individualism–collectivism dichotomy, and of its relationship with human social behavior and decision making. Other proposals, such as the GLOBE model have expanded Hofstede’s initial dimension, with more detailed distinction in the individualism–collectivism dichotomy, differentiating between institutional vs. in-group collectivism (House et al., 2004). Moreover, Pelham et al. (2022) have recently developed a Global Collectivism Index, more comprehensive that the one created by Hofstede decades ago. This new index encompasses data from 188 countries, a substantial expansion compared to Hofstede’s previous classification of 69 nations.

In individualistic cultures, personal needs are prioritized over the groups and individuals value independence, autonomy, and self-reliance (Triandis, 2001). Conversely, in cultures with a collectivist orientation, individuals prioritize interdependence, recognizing the interconnectedness of the self, and emphasizing the importance of relationships. It follows that the societal values of individualism and collectivism significantly can impact a country’s approach to balancing individual freedom and collective/public good (Conway et al., 2006). Additionally, Hofstede’s cultural dimensions of power distance, uncertainty avoidance, and indulgence vs. restraint may also influence these outcomes (Hofstede, 2001). Power distance, as a cultural dimension, encompasses the degree to which a community acknowledges and approves of authority, power differentials, and status privileges (Carl et al., 2004). Italy aligns with nations exhibiting a higher tolerance for power distance, therefore, in the Italian context, there is an acceptance and, to some extent, an expectation that certain societal groups hold greater power than others (Tavanti, 2012). Uncertainty avoidance is defined as the extent to which people feel threatened by uncertain or unknown situations (Hofstede, 2011). Italy shows a high level of Uncertainty Avoidance Index, compared to the other Northern European countries. The dimension of Indulgence vs. restraint is defined by the degree to which individuals seek to manage their desires and impulses. “Indulgence” characterizes relatively weak control, while “Restraint” signifies strong control. Italy’s low score in this dimension denotes that Italian culture leans towards Restraint.

According to this literature, both Southern Mediterranean and Scandinavian countries, although differing on many other cultural features, seem to be characterized by similar levels of individualism–collectivism. Anyway, Italy exhibits a greater inclination towards collectivism (ranked 150 out of 188) compared to Norway (182) and Sweden (187) in the Pelham et al. classification (2022), from the most collectivistic to the least.

About collectivism, it can be distinguished in horizontal and vertical (Triandis and Gelfand, 1998). Horizontal collectivism, also known as equality-oriented collectivism, is marked by an emphasis on egalitarianism, shared responsibilities, and group harmony. In societies exhibiting horizontal collectivism, individuals are considered equals, and there is a collaborative spirit that discourages significant hierarchical structures. Cooperation and mutual support are valued, fostering a sense of interconnectedness among individuals. On the other hand, vertical collectivism, or hierarchy-oriented collectivism, places importance on social hierarchies, status differences, and respect for authority. In cultures with vertical collectivism, individuals accept unequal power distribution and distinct social roles, with an emphasis on maintaining group unity through a structured hierarchy.

When it comes to measures related to the COVID-19 pandemic, it has been argued that in collectivist societies, people would expect leaders to prioritize the well-being of the group over individual freedom. On the other hand, in individualistic cultures, individuals’ well-being would likely be given more importance over the well-being of the group (Bajaj et al., 2021).

Several social factors, including gender (Galasso et al., 2020), trust (Gennaro et al., 2023), and political affiliation (Gollwitzer et al., 2020), have been shown to influence attitudes, behaviors, and health outcomes related to the pandemic. Among these factors, cultural orientation in the form of individualism–collectivism has received significant attention in academic research (Ashraf et al., 2022). While previous studies primarily have focused on how individualism–collectivism affects behavior, such as mobility reduction, social distancing, and mask usage (Lu et al., 2021), this study seeks to explore whether cultural factors may have influenced leaders’ decision-making during the pandemic. Through a qualitative analysis of interviews with key political-administrative leaders, we aim to identify the explicit and implicit cultural dynamics that might have played a role in these decisions.

Previous research has established a connection between individualism/collectivism and acceptance of authority, including government-imposed restrictions. For instance, in cultures that value individualism, the government is less likely to enact policies that infringe on citizens’ personal rights and freedoms, and may face challenges in implementing and enforcing mobility restrictions to combat the spread of COVID-19 (Lu et al., 2021; Huang et al., 2022). This is due to the reluctance of individuals to comply with such restrictions and the difficulty of enforcing them, potentially leading to a more severe outbreak. On the other hand, collective attitudes are linked directly to increased cooperation and compliance with preventive measures, and more compliance with rules and government regulations (Maaravi et al., 2021). For example, a collectivistic approach to address the COVID-19 pandemic was characterized by implementing mask mandates for the safety of the general population. Conversely, in other countries such as Sweden, mask mandates were not put in place, and individuals were expected to rely on their own immune systems (Giritli Nygren and Olofsson, 2021).

Therefore, policymakers should not only focus on technical measures. It is likely that they develop the Covid response measures by reinforcing them with cultural elements; considering the cultural factors that are specific to their country when making policy choices (Gokmen et al., 2021).

Communication also should reflect the culture to increase the probability of compliance with the measures. For example, in collectivistic cultures, a communication strategy based on protecting others may work better (e.g., “maintaining physical distancing can protect you and your family from infection”), whereas in individualistic cultures, a communication based on self-protection (e.g., “maintaining physical distancing can protect you from infection”) might be more effective (Gokmen et al., 2021). It may be that Sweden, as a rather individualistic culture, did not enforce strict mobility restrictions during the pandemic. Instead, they relied on individuals to take personal responsibility and follow guidelines, which led to a relatively high number of COVID-19 cases and deaths compared to other countries. Sweden’s response of rejected lockdown policies was possible because of the high level of trust between the individuals and the state (Johansson et al., 2021). Norway followed a path similar to Italy and other European countries by implementing measures such as school and business closures, strict travel restrictions, and more (Christensen and Lægreid, 2020).

## 2 Method

This study aims to explore the potential relationships between individualism–collectivism values and policy-making across countries. We do not propose specific predictions about the direction or strength of these relationships, but we seek to identify and interpret meaningful patterns and trends through qualitative analysis. Our goal is to uncover insights into how cultural dimensions may have interacted with policy development. We conducted interviews with key political administrative leaders in Italy, Norway and Sweden to gather their insights on the first phase of the pandemic, which spanned approximately from January to July 2020. The current brief report presents a preliminary analysis of interviews conducted in Italy, to investigate the cultural dynamics and analyze the role of individualism and collectivism in the decision-making process during the pandemic.

The 35 Italian interviews include actors in the health sector (*N* = 10), committees of experts from different fields (e.g., medical, economic, political) (7), Department of Civil Protection (7), regional policy making (4), educational sector (5), Diplomatic Corp (2). Participants were reached out to through email or personal contact, selected based on their roles during the initial phase of the pandemic. Involvement in the study was entirely voluntary; consequently, some individuals we approached for interviews were unavailable to partake in the study. Interviews were concluded once the topics reached saturation.

Interviews were audio and video-recorded (when conducted online) and then transcribed verbatim and content-analyzed using a qualitative approach. The methodology employed for this study was rooted in the grounded theory approach, in its earlier formulations (e.g., Glaser and Strauss, 1967) and later refinements (e.g., Strauss and Corbin, 1990) following a more constructivist approach to Grounded Theory (e.g., Charmaz, 2006). The research design embraced a bottom-up perspective, allowing themes and patterns to emerge organically from the data, and coding and interpreting them according to our initial discussion of the individualism–collectivism dichotomies proposed by various authors. The content analysis was conducted manually, with emerging themes identified and compared through in-depth discussions. No specific software was employed in the qualitative analysis. To ensure quality control and consistency in interpreting the transcripts, two independent researchers performed the initial analysis, and any discrepancies were resolved through consensus discussions (Flick, 2004). As a general methodological guideline, we followed the COREQ Checklist and made sure to respect its main principles (Tong et al., 2007).

This study has been conducted in full compliance with the national ethical guidelines of Italy, in particular we adhered the ethical code for research in psychology defined by the Italian Psychological Association. This study is also part of an international project that was approved and funded by the Norwegian Council of Science and, as such, its ethical aspects were acknowledge by the funding agency. All participants were assured of the anonymous character of the interviews and provided informed consent prior to their participation in the study.

## 3 Results

The COVID-19 pandemic, which originated in China, rapidly spread to other countries, with Italy becoming the first and most severely hit point of outbreak in Europe. Italy experienced one of the highest death tolls in the world^1^, particularly in the Lombardy region, which was severely impacted by the pandemic, especially the healthcare system.

In Italy, the government did take several measures that seem to reflect collectivist values, such as imposing strict lockdowns and restrictions on movement to slow the spread of the virus. These measures were aimed at protecting the entire population, even though they had a significant impact on individuals and the economy. The Italian government’s approach to the pandemic apparently reflected a collectivist mindset, putting the well-being of society over individual interests. But how was this decision made? What considerations were taken into account?

In making decisions during the first months of the pandemic, the Italian Government sought advice of many different expert boards and committees, such as a Scientific-Technical Committee (*Comitato Tecnico-Scientifico; CTS*). A member of one of those committees, reported how making the lockdown decision was based first on medical advice to contain the spread:

*And the only way to interrupt the circulation was to stop everything. So, we realized in that moment […] so our advice* (to the Government) *was “you need to stop everything, because this is technically the only way to solve the diffusion of the pandemic, it is to stop everything.” That was a medical advice because with all the deaths… we analyzed the problem from the medical perspective, and we gave indication.*

The government also prioritized those measures that would protect the vulnerable (such as the elderly, who are at higher risk of severe illness and death from COVID-19). In this extract the interviewee told us how the lockdown was a way to protect the people most at risk, despite the great sacrifice for the population:

*When we started to realize the problem and we started to close ten municipalities in Lombardy, we started to realize the real meaning of lockdown. And the real meaning was, we have to take care of the people that are living there, the need of a lot of people, the need of people with emergency needs, the need of children and elderly, sick people, essential business, essential work, the people who were obliged to move and to go to the market to work or different…*

One of our interviewees also reported that in making this decision, they had no idea how the Italians would react, as they were concerned about the social character of the Italians.

*…lockdown was a real and new business in our culture. So, let us say that we were not culturally prepared. […] We were really much worried to see the reaction of the Italians, because we know our country*.

Nevertheless, the Italian citizens – perhaps reflecting a value of collectivism in which the good of the group and the population most at risk is a priority over the individual – followed the lockdown instructions without protesting too much.

**Figure 1:**
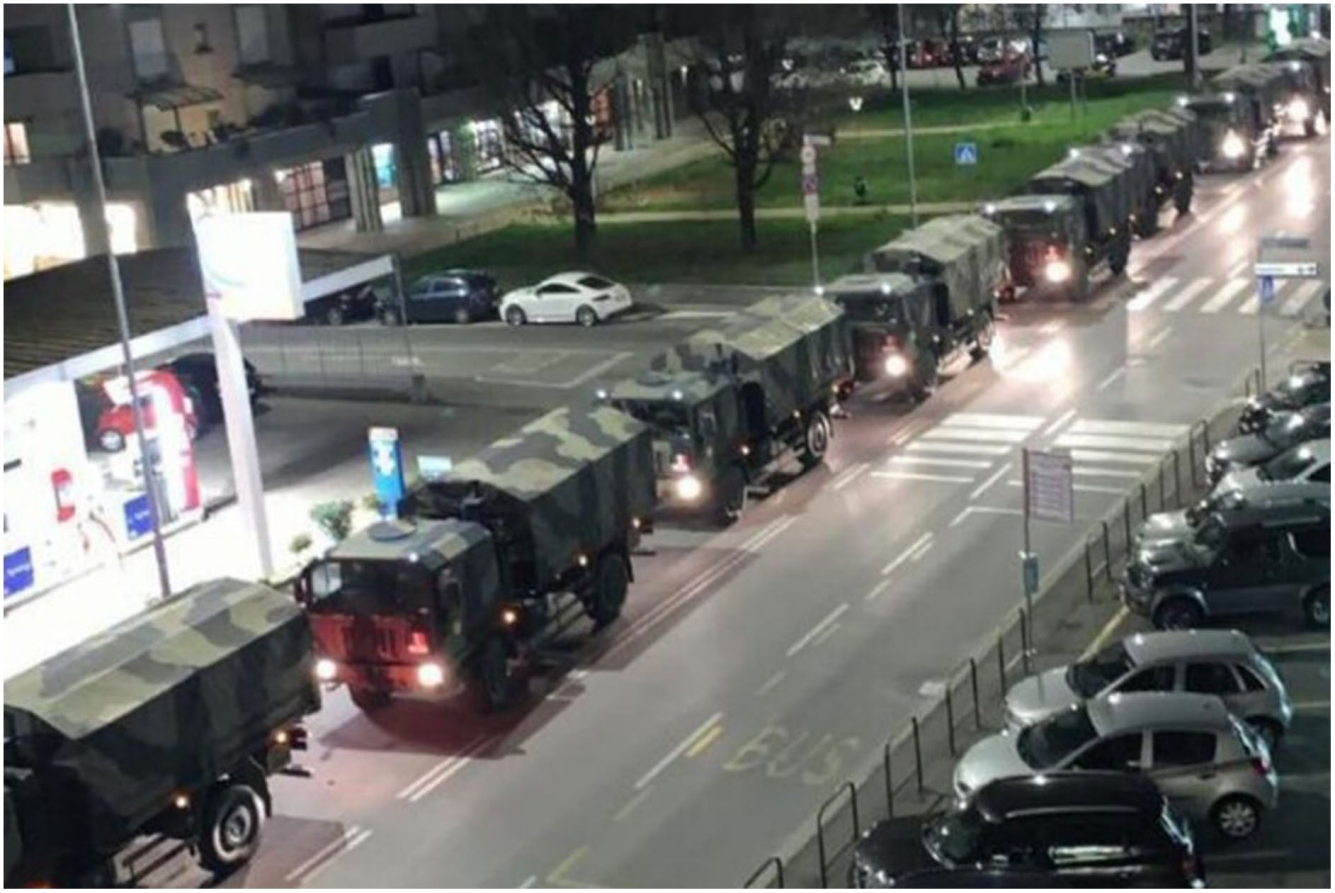
Shocking picture of military trucks taking away corps in Bergamo, Lombardy, March 19th, 2020. Source and credits: ANSA.

The first moment when most people realized the seriousness of the situation, which then prompted the government to issue a national lockdown, was when a picture (see Figure 1) circulated of military vehicles taking away coffins because the civic funeral service was unable to cope with the huge number of dead.

This exact incident is what made the situation clear, made people more aware of what was happening, and triggered fear in the citizens, but more importantly, increased responsibility toward others. This episode was reported to us by most of the interviewees, and it had consequences for people’s decisions and actions. Here’s some examples:

*People looked at thousands of coffins brought away by military trucks. That gave to all Italian citizens a real taste of what covid was. One of the richest parts of Italy, one of the best, equipped with thousands of people dying, and so many coffins that had to involve the military transportation trucks to bring them away from... And that was one of the motivations that clearly helped us to make people understand that the lockdown was necessary, and we were the first democratic country to lockdown a whole country for two months, and people complied*.

*…an historical picture, that you know, sometimes pictures are the triggers for decision. Our military trucks that were taking the dead bodies because they were unable to take care about the dead’s*.

*We had at a certain moment several military trucks – and there is an historical picture with twenty tracks – full of dead bodies from the hospital in Bergamo and that region. It was the sign, the physical sign that the situation was already over and above our capacity to control.*

Italy’s collectivist cultural values also were reflected in hospitals, typically an individualist context (De Bono et al., 2014). For example, hospitals’ leaders explained how they adapted entire wards of the hospital so that the people working inside could have a bed to sleep in and not go home for fear of infecting their families:

*…for a few days the staff, for example, of the medicine where there were at least a dozen suspect patients who later turned out to be mostly infected with covid, they no longer wanted to change shifts. They did not want to go home. They were so afraid… [….]. Objectively, there was a situation in which for different reasons they did not accept the shift change. They stayed in the ward and did not want to go home anymore. Just as there were people who were at home who no longer wanted to come to the hospital*.

*And some people stopped going home […] they started living in the hospital or just outside. We have also set up spaces for those who did not want to go home, for those who were afraid of infecting their families and wanted to stop and sleep, we had some administrative spaces that we transformed by putting cots, activating bathrooms*.

In Italy, all messages about Covid and safety were aimed at educating the entire population about the virus, its symptoms, and how to protect themselves and others. This approach reflected the value of protecting the well-being of a society as a whole, even if it meant making sacrifices or placing restrictions on individuals. The campaigns were designed to promote a sense of collective responsibility, encouraging people to take actions that would protect not only themselves, but their families, communities and especially the health care professionals who were overwhelmed as well. A member of the Civil Protection told us how they spread this message in the local communities:

*Even in situations where a person can be a little bit more emotional - and I personally am very sensitive, and I am very sorry to see these things - the message was “close your eyes” and be strong to help those who were weak at that moment. This is a message we gave ourselves and everyone. [...] With one of our service cars we went out during the lockdown and with a recorded voice message we advised everyone not to go out.*

The Italian government’s public information campaign about COVID-19 was approached with a collective mindset as well, promoting the idea that “we are all in this together.” The Prime Minister’s role in this campaign was that of a leader who cared for all the Italian people, much like a head of a family. This sentiment was reflected in the feedback of interviewees who described the experience as one of a united and collective effort to overcome the pandemic:

*So, initially it was – let us say, initially it was almost reassuring communication, the early days. Of the series: yes, there is an emergency, but we are structured here in such a way that... there is a health system that works very well*.

*So that’s why I’m saying that’s what was expected of [President] Conte to take very strong personal leadership. And the conversation and the “trust me, I will take care of you.”*

So*, in a way I mean you got the communication from [President] Conte, that was kind of the father figure, and then you had the local leadership really imposing measures on you. And then in reality, “andrà tutto bene”* [Ndr. Italian slogan used during the pandemic meaning “everything will be ok”] *at the end, if we just stick together and do what we know is best for ourselves as a family or for the community we are living in. So, you had a lot of community activities. I guess people were helping each other, buying food for the old woman in the doorway. All this kind of community feeling, that was non-governmental or not initiated by authorities but a kind of collective awareness of wanting to do good*.

The final message that came through was, as a member of the diplomatic corps reported:

*Do it for the others, for your own family, for the community. And I think that the sense of community got stronger*.

The value of collectivism in decision-making in Italy is encapsulated in an extract of an interview with a member of one of the many advisory committees. The pandemic being a social crisis as well as a health crisis, it meant that the ultimate goal of “caring” for a community, rather than for individuals was at the heart of every decision. This principle that guided every decision made and all advice given is exemplified in this sentence:

*Most probably what happened in that period was exactly this. We were prepared not to answer to this specific emergency that was the corona virus. We were prepared to answer as community. So, we were prepared to move on, to react, to take care. The way we answered to the lockdown, the way we answered to other difficult decisions, to stop school, to close the school, to close the football games*.

## 4 Discussion

The focus of this report was on the Italian process of decision-making and how the country’s individualistic-collectivistic values influenced decision-making and actions during the COVID-19 pandemic. Italy experienced a significant outbreak of the virus, with one of the highest death tolls in the world, particularly in the hard-hit Lombardy region. The government implemented strict lockdown measures and restrictions on movement (Hale et al., 2020) in an effort to slow the spread of the virus and protect the entire population, even though these measures had a significant impact on individuals and the economy. The qualitative analysis presented in this paper offers a nuanced perspective on the government’s approach. Despite Italy typically being labeled as a predominantly “individualistic” nation, our findings suggest that the decision-making process may have been influenced by a more collectivistic mindset among its leaders. Several factors might have contributed to this change. First and foremost, the emphasis on family and communal ties in addressing the challenges of isolation may have played a crucial role. This emphasis likely heightened the sense of collectivism among citizens during the lockdown (Travaglino and Moon, 2021; Gennaro et al., 2023). This shift reflects a noteworthy departure from the conventional characterization of Italy as an individualistic society. Notably, Italian government officials, experts, and professionals in charge of leading the country’s first response to the Covid-19 pandemic, in which the well-being of society as a whole was prioritized over individual interests. Also, the willingness of Italians to comply with lockdown measures, the sense of community support, and the adoption of collective responsibility can be recognized as reflections of collectivistic values. This is consistent with previous research that has demonstrated a positive association between heightened collectivism and increased support as well as adherence to COVID-19 preventive measures (Travaglino and Moon, 2021; Pei et al., 2023).

Understanding the influence of these cultural values can provide valuable insights for policymakers and public health authorities (Nair et al., 2022) when addressing future public health challenges, in particular when it concerns the public acceptance or rejection of specific public measures (Bavel et al., 2020).

In acknowledging the limitations of our study, it is essential to note that the selection of Italy, Norway and Sweden was dictated by the parameters of the overall CORNER project. Although these countries show a predominant trend toward individualism on the individualism–collectivism scale, we recognize the limitation in capturing a more diverse range of cultural variation within the selected countries. It would be interesting to compare in further studies, countries with distinct levels of individualism–collectivism, such as Western and Eastern European nations.

Another limitation, of course, is dictated by the condensed nature of this study being a short report with a focus on one country out of three, which does not allow for a greater exploration of the factors considered. Nevertheless, this preliminary analysis shows why conducting cross-country analyses of the COVID-19 response may offer valuable insights about how collectivistic and individualistic values shape decision-making, societal responses, and outcomes in global challenges. The different approaches adopted by different countries, combined with their shared cultural emphasis on community or individual well-being, provide a compelling framework for examination.

By exploring the different strategies employed, ranging from strict lockdown measures in Italy and Norway to a more relaxed approach in Sweden, we can explore their alignment with cultural values. Italy’s experience of a severe outbreak and high death toll, coupled with a collectivistic approach, provides a meaningful reference point for comparison. Examining public perception and compliance with pandemic measures in each country highlights the interplay between cultural values and individual behavior. Understanding how collectivistic values influence decision-making and public response in Italy, Norway, and Sweden may contribute to a more comprehensive understanding of societal dynamics during global challenges. The variation in outcomes across these countries also provides critical insights into the impact of different strategies and their effectiveness in mitigating the spread of the virus, while protecting public health. These insights can inform future policymaking and crisis management efforts, enabling the development of more effective strategies that align with cultural values and promote collective well-being, ensuring that cultural values are integrated into decision-making processes that promote more cohesive and resilient societies.

## Data availability statement

The raw data supporting the conclusions of this article are not publicly available due to concerns regarding participant anonymity. Requests to access the datasets should be directed to the corresponding author.

## Ethics statement

The studies involving humans were approved by the Norwegian Council of Science and ethical approval was obtain by the ethics committee of Roma Tre University. The study was conducted in accordance with the local legislation and institutional requirements. The participants provided their written informed consent to participate in this study.

## Author contributions

SC: Writing – original draft, Writing – review & editing, Conceptualization, Formal analysis, Investigation. GC: Conceptualization, Supervision, Writing – original draft, Writing – review & editing, Funding acquisition.

## Footnotes

^1^ https://covid19.who.int/region/euro/country/it
